# Childhood Obesity among Puerto Rican Children: Discrepancies Between Child’s and Parent’s Perception of Weight Status

**DOI:** 10.3390/ijerph9041427

**Published:** 2012-04-16

**Authors:** Winna T. Rivera-Soto, Linnette Rodríguez-Figueroa

**Affiliations:** 1 Department of Human Development, Graduate School of Public Health, University of Puerto Rico, Medical Science Campus, San Juan, PR 00936-5067, Puerto Rico; 2 Department of Biostatistics and Epidemiology, Graduate School of Public Health, University of Puerto Rico, Medical Science Campus, San Juan, PR 00936-5067, Puerto Rico; Email: linnette.rodriguez3@upr.edu

**Keywords:** Puerto Rican childhood obesity, parental perception, children perception

## Abstract

Public concern about childhood obesity and associated health problems calls for the identification of modifiable factors that could halt this epidemic. Parental perceptions of their children’s weight status could be associated to how parents influence children’s eating patterns. We aimed to identify the perceptions Puerto Rican parents have of their children’s weight and children’s own perceptions of weight status as compared to real weight. A cross sectional survey was performed in a representative sample of 1st–6th grade students. Only half of the children correctly identified their weight, and only 62.4% of the parents correctly classified their children’s weight. Most obese/overweight children did not perceive themselves as such. Almost half of obese/overweight children were identified by the parents as normal weight while over half of the underweight children were perceived by their parents at normal weight. More girls than boys perceived themselves as obese/overweight and more parents of girls than of boys perceived them as such. Higher-educated parents were better at recognizing overweight/obesity among their children compared to less-educated parents. This study suggests an influence of parents’ SES characteristics on their perceptions of children’s weight status as well as on children’s own perceptions of their weight status.

## 1. Introduction

Public health concerns about childhood obesity and its association to health problems, both short term (in childhood) and long term (in adulthood), have led to the identification of modifiable factors that could halt this epidemic. Childhood obesity stems from the interplay of multiple influences; however, parents are among the most influential factors in laying the foundation for early childhood weight problems [1]. Studies confirm the influence that parents exert over children’s dietary and lifestyle practices, impacting their weight and nutritional status. A meta-analysis study by Golan and Crow [2] describes the role of parents in the etiology and prevention of weight-related problems as role models, and through determining the home environment. Caregivers directly determine a child’s lifestyle, environment and body weight through food selection, home eating patterns, meal structures, responsiveness to child’s feeding cues and general parenting styles [3]. Children raised in authoritative homes tend to eat more healthily, are more physically active, and have lower BMI levels compared to children raised with other styles, such as more permissive/indulgent environments [4].

Parents provide food environments for their children’s early experiences with food and eating [5]. Family eating environments include the parents’ own eating behaviors and child-feeding practices. In particular, parents who are overweight, who have problems controlling their own food intake or who are concerned about their children’s risk for being overweight may adopt controlling child-feeding practices in an attempt to prevent overweight in their children. A study by Sealy [6] showed how food choices of parents and children are closely connected with culturally-determined eating habits. Caribbean participants differ from both African American and Puerto Rican participants in their attitudes and practices regarding food [6,7,8].

Parents’ influences may be associated to perceptions of their children’s weight status. A large percentage of parents do not perceive their children to be overweight and are not concerned about health risks [9,10]. Parents of obese and overweight children tend to underestimate their child’s weight status compared with underweight/normal-weight children [11,12,13,14]. On the other hand, parents of boys are more likely to perceive their child’s weight incorrectly compared to parents of girls [15].

Socio-economic factors have also been identified as a strong factor in the underestimation of children’s overweight or obesity [16]. Mothers with a lower educational background are more likely to misclassify overweight silhouettes and underestimate overweight-associated health problems [17]. There was an inverse relationship between the mothers’ ability to accurately predict their child’s body weight and maternal educational level. More mothers with lower educational levels failed to perceive their children as overweight compared to mothers who completed high school or some college education (30% *vs.* 17%).

Discrepancies among children’s and parents’ perceptions of children weight status seem to be associated to parents’ weight status. A meta-analysis conducted by Doolen *et al*. [12] concluded that parents who were themselves overweight were more likely to misperceive their child’s overweight. Overwhelmingly, these researchers found that the majority of parents, specifically mothers (only two studies acknowledged fathers), failed to accurately perceive the weight of their obese or overweight child. Regardless of the child’s age, this tendency seems consistent across studies that studied parental perceptions throughout various countries. A study among Australian mothers showed problems recognizing their children’s overweight status. The prevalence of overweight or obesity was 19%; however, only 5% of Australian mothers indicated concern about their child being currently overweight [18]. Myers and Vargas [19] surveyed 200 parents of overweight US children in a WIC program; approximately 95% of participants were Hispanics and the other 5% were African Americans, Africans, or Asians. Forty-three percent of children in this study were obese. However, only 7% of parents accurately recognized their child as overweight. A recent review conducted by Ward [20] showed the tendency of Mexican mothers of failing to accurately perceiving their children’s overweight. However, in our literature review we were unsuccessful to find a study about perceptions of Puerto Ricans parents of their children’s weight status, the second largest Hispanic population in the United States.

In an effort to measure prevalence of childhood obesity among Puerto Rican children and associated factors, a research study was conducted in a suburban municipality of Puerto Rico among elementary school children (first to sixth grade) from private and public schools [21]. The study aimed at examining the association of dietary, socioeconomic and environmental factors to children’s weight status. A high prevalence of childhood obesity (26.8%) was found in this group [21]. This prevalence was higher than the ones reported by Ogden *et al*. [22], for US overall children (19.6%) or US Hispanic children (25.1%).

The present article focuses on describing the association between parents’ perceptions of their children’s weight status and children’s real weight status. In addition, it explores the association between children’s real weight status and perceptions of their own weight status. These analyses are particularly needed given the high prevalence of childhood obesity found in the island and the need to better address influencing factors such as family environment. Moreover, no previous studies have explored perceptions of Puerto Rican parents regarding their children weight status.

## 2. Methodology

This study adopted a cross-sectional randomized descriptive design. Previous report provides details of study design and sampling frame [21]. For the purpose of this article we will focus on methods regarding the measurement of parents and children’s perceptions of children’s weight status. A validated and self-administered questionnaire, along with a consent form for participation, were sent to parents through homeroom teachers. This questionnaire included questions on family’s socio-demographic and socio-economic characteristics and parents perceptions of children’s weight status, among others. Family socio-economic status (SES) was measured by means of two variables: family monthly income and health insurance coverage. This last criterion was used as a proxy of socioeconomic status since eligibility for participation in the public health insurance, called “La Reforma de Salud” (Health Reform), is based on poverty level. Parents returned the questions to the teachers in a sealed envelope provided by the researchers.

A trained interviewer administered the children’s questionnaire at the school. The questionnaire included questions regarding dietary practices, physical activities and leisure activities, as well as children’s perception of their own weight status. Weights and heights were measured to the nearest 0.1 kg and 0.1 cm, respectively, by survey personnel using a portable and standardized scale/stadiometer (*Detecto*). Shoes were removed for the height and weight measurements. All measurements were taken twice by the same research staff member. Children’s weight status was determined based on the 2000 sex-specific body mass index BMI-for-age growth charts from the Centers for Disease Control and Prevention [23]. Briefly, children at or above the 95th percentile were defined as obese; those above 85th and below 95th percentiles were defined as overweight; children under the 85th percentile and above the 5th percentile were classified as normal weight and those under the 5th percentile were defined as underweight. The BMI of the parents was estimated using their self-reported height and weight.

Data were analyzed using SPSS (*Statistical Package for the Social Sciences*) for Windows (version 15.0), and SAS (*SAS Institute Inc*.) for Windows (version 9.1). Chi-square or Fisher tests were used to compare proportions. Kendall’s Coefficient of Concordance (W) was estimated to test agreement among the respondents, where W = 0 signifies no overall trend of agreement. This sample was representative of all students between first and sixth grade in Cayey. Results were weighed using the inverse of the probability of sample selection [24]. All analyses were performed on weighed data.

## 3. Results

A total of 250 students and their parents participated in the study. This represented a response rate of 63.0% among children and 44.0% among parents. Nearly half of the students (51.0%) were boys and the mean age was 9.5 y/o; 78.1% were from public schools; 52.7% were located in a rural area; and 52.2% had government-provided health insurance “*Mi Salud*” (data not shown). Approximately 38.1% of the children were classified as obese or overweight and 5.7% as underweight (Table 1). On the other hand, 27.6% of parents were classified with some type of overweight, whereas 36.7% were underweight. Despite of the high prevalence of childhood obesity/overweight in the sample (38.1%), only 20.0% of parents classified their children as such, and only 20.3% of the children considered themselves overweight/obese. Approximately 5.7% of all children were underweight; however, 17.3% of the parents perceived their children to be underweight and 23.1% of children perceived themselves underweight.

**Table 1:** Estimates of Real and Perceived Weight Status among Elementary School Students and their Parents, Cayey, Puerto Rico, 2008.

Weight status	Obese or overweight n (%)	Normal weight n (%)	Underweight n (%)
Child’s real weight (BMI for age/sex)	92 (38.1)	136 (56.2)	14 (5.7)
Child’s self-perceived weight	45 (20.3)	125 (56.6)	51 (23.1)
Parent’s perception of child’s weight	41 (20.0)	127 (62.7)	35 (17.3)
Parent’s real weight (BMI)	31 (27.6)	40 (35.7)	41 (36.7)

The prevalence of the child’s weight status was marginally different (*p* = 0.055) by gender: more females were classified as obese or overweight (39.7% *vs.* 36.4%) and underweight (9.1% *vs.* 2.5%) compared to males (Table 2). Perceived weight (both parent’s and child’s) were statistically similar by gender, although there is a tendency for more female than male children to perceive themselves as obese or overweight (22.2% *vs.* 18.3%), and for more parents of female than male children to perceived them so (23.8% *vs.* 16.7%). Real and perceived weight status was also statistically similar by type of school (public or private) and by parent’s educational level.

**Table 2:** Estimates of Real and Perceived Weight Status by Child’s Gender, School Type, and Parent’s Educational Level among Elementary School Students, Cayey, Puerto Rico, 2008 *.

Child’s weight status	Real weight (BMI for age/sex) *	Perceived weight (by child)	Perceived weight (by parent)	Real weight (BMI for age/sex) *	Perceived weight (by child)	Perceived weight (by parent)
	n (%)	n (%)	n (%)	n (%)	n (%)	n (%)
	**Female (n = 122)**			**Male (n = 128)**		
Obese or overweight	48 (39.7)	24 (22.2)	24 (23.8)	44 (36.4)	21 (18.3)	17 (16.7)
Normal weight	62 (51.2)	62 (57.4)	63 (62.4)	74 (61.2)	64 (55.7)	64 (62.7)
Underweight	11 (9.1)	22 (20.4)	14 (13.9)	3 (2.5)	30 (26.1)	21 (20.6)
	**Public school (n = 195)**			**Private school (n = 55)**		
Obese or overweight	70 (37.6)	36 (21.7)	31 (20.0)	22 (40.0)	9 (16.4)	9 (18.8)
Normal weight	105 (56.6)	95 (57.2)	98 (63.2)	30 (54.5)	30 (54.5)	30 (62.5)
Underweight	11 (5.6)	35 (21.1)	26 (16.8)	3 (5.5)	16 (29.1)	9 (18.8)
	**Parents with high school graduate or less (n = 76)**			**Parents with some after high school (n = 144)**		
Obese or overweight	25 (34.2)	13 (20.0)	11 (16.9)	52 (36.9)	28 (21.5)	30 (22.9)
Normal weight	44 (60.3)	40 (61.5)	42 (64.6)	79 (56.0)	64 (49.2)	79 (60.3)
Underweight	4 (5.5)	12 (18.5)	12 (18.5)	10 (7.1)	38 (29.2)	22 (16.8)

* Comparison of real weight by gender: *p* = 0.055. All other comparisons: *p* > 0.05.

Only half (50.9%) of the children correctly identified their weight and only 62.4% of the parent’s correctly classified their children’s weight (Table 3). Most of the obese or overweight children did not perceive themselves as such: 10.3% perceived themselves as being underweight and almost half (48.7%) as having normal weight. Also, a high percentage of the underweight children (78.6%) perceived themselves as having normal weight. Moreover, almost half (48.6%) of obese or overweight children were identified by the parents as being normal weight. In addition, over half (53.8%) of the underweight children were perceived by their parents as being in a normal weight.

**Table 3:** Relationship between Real and Perceived Weight Status among Elementary School Students, Cayey, Puerto Rico, 2008.

Perception of child’s weight status	Child’s real weight (BMI for age/sex)		
	Obese or overweight n (%)	Normal weight n (%)	Underweight n (%)
Perceived by child*			
Obese or overweight	32 (41.0)	9 (7.4)	-
Normal weight	38 (48.7)	74 (60.7)	11 (78.6)
Underweight	8 (10.3)	39 (32.0)	3 (21.4)
**Perceived by parent^†^**			
Obese or overweight	37 (50.0)	2 (1.8)	-
Normal weight	36 (48.6)	80 (72.7)	7 (53.8)
Underweight	1 (1.4)	28 (25.5)	6 (46.2)

* Only 50.9% of concordance between the real and perceived weight. Kendall’s W = 0.084 (*p* < 0.001); ^†^ Only 62.4% of concordance between the real and perceived weight. Kendall’s W = 0.124 (*p* < 0.001).

Parental perceptions may be influenced by parents’ own weight status (Table 4). In general, classification of children’s weight by their parents tended to correspond to their parents’ weight status, except for underweight parents. Approximately 68% of obese parents who had obese children correctly classified their children as such and 75% of normal weight parents with normal weight children correctly classified their children weight status. Only 25% of underweight parents with underweight children correctly classified their children. On the other hand, a considerable percentage of obese parents (31.8%) failed to correctly identify obesity in their children. In addition, more than two thirds (66.7%) of obese parents with normal weight children misclassified their children as underweight. This tendency was also seen among normal weight parents: 54.5% misclassified their obese children as normal weight. In addition all (100%) of the underweight parents who had obese children misclassified their children as normal weight.

**Table 4:** Relationship between Child’s Real and Perceived Weight Status by Parental Weight Status among Elementary School Students, Cayey, Puerto Rico, 2008.

Parent’s real weight (BMI)	Parent’s perception of child’s weight	Child’s real weight (BMI for age/sex)		
		Obese or overweight n (%)	Normal weight n (%)	Underweight n (%)
Obese or overweight * (n = 28)	Obese or overweight	15 (68.2)	-	-
	Normal weight	7 (31.8)	2 (33.3)	-
	Underweight	-	4 (66.7)	-
Normal weight ^† ^(n = 38)	Obese or overweight	10 (45.5)	2 (12.5)	-
	Normal weight	12 (54.5)	12 (75.0)	-
	Underweight	-	2 (12.5)	-
Underweight ^†† ^(n = 40)	Obese or overweight	-	-	-
	Normal weight	4 (100.0)	18 (56.3)	3 (75.0)
	Underweight	-	14 (43.8)	1 (25.0)

* Only 60.7% of concordance between the real and perceived weight. Kendall’s W = 0.131 (*p* = 0.035); ^†^ Only 57.9% of concordance between the real and perceived weight. Kendall’s W = 0.003 (*p* = 0.715); ^††^ Only 47.5% of concordance between the real and perceived weight. Kendall’s W = 0.332 (*p* < 0.0001).

## 4. Conclusions

This study evidences the high prevalence of overweight among Puerto Rican children and the failure of both parents and children themselves to correctly assess children’s weight status. We found significant discrepancies between children’s real weight status and perceptions among parents and children themselves of children’s weight, particularly for obesity/overweight.

Parents of overweight/obese girls were more accurate in perceiving their child’s weight status compared to parents of overweight/obese boys. This tendency needs further attention as males tend to have a higher prevalence of obesity-related conditions, particularly cardiovascular diseases [25], at a younger age compared to women. On the other hand, parental recognition of girls’ overweight/obesity status represents a good opportunity for parental and child education on the prevention of obesity-related conditions more prevalent among women, such as breast cancer [26,27]. An elevated underestimation of overweight was also observed among overweight parents. However, it was more evident among parents of boys compared to parents of girls.

Consistent with previous studies, certain socio-economic characteristics among parents seem to predict these discrepancies. There was a tendency among less-educated parents to underestimate their children’s overweight/obesity status. A higher percentage of parents with more than a high school education tended to recognize children’s overweight or obesity than parents with less than a high school education. These tendencies were also seen among their own children. A greater percentage of children of higher-educated parents correctly estimated their own overweight status.

This study also shows that a higher percentage of parents of children from private schools tended to underestimate their children’s weight status compared to parents of children in public schools, particularly regarding obesity or overweight. Similarly, parents of children from private schools tended to overestimate underweight among their children. These contradictory findings are of concern since children from private schools tend to come from families with higher income levels, and therefore have access to more economic resources to purchase food at the school cafeteria. Despite the fact that in Puerto Rico competitive foods are prohibited by law to be sold in schools and nearby environments, these products could still be available for sale in fund-raising activities within the school setting.

Girls themselves classified their weight more correctly than boys. A higher percentage of obese/overweight boys misclassified themselves as normal weight compared to girls. These findings could be associated to the fact that mass media campaigns are targeted to women’s weight control compared to men. On the other hand, it rises up a concern of whether men are less concerned about overweight/obesity problems, thus increasing their chances of developing associated chronic diseases early in life.

A noteworthy contribution of this study is the analysis of the concordance between children’s real weight compared to parents’ and children’s perceptions of weight status. In general, we found that only half of children and their parents were in agreement with each other regarding weight classification. These discrepancies in perception were even more notable among overweight/obese subjects. Only half of the parents of overweight children correctly perceived their children’s overweight status. The other 50% of parents of overweight/obese children considered their children to be at a normal weight. A considerable percentage of children who perceived themselves as overweight were perceived by their parents as being of normal weight. The same tendency was seen among overweight children. In general they leaned to underestimate their weight status. Almost half of overweight children considered themselves to be at a normal weight.

Parents of underweight children also were inclined to misclassify their children’s weight. Less than half of parents of underweight children recognized the weight status of their children. On the other hand almost two thirds of children and their parents perceived the children to be in normal weight whereas only one third of both parents and children perceived children to be underweight.

Parents’ own weight status seems to play an important role on their perceptions of children’s weight status. In general, parental weight status seemed to influence the parent’s classification of their child’s weight status. However, there were some exceptions. A considerable percentage of both overweight and normal weight parents underestimated their child’s overweight status. However, to our surprise, this tendency was higher among normal and underweight parents. A higher percentage of normal weight parents were unable to classify their child as overweight compared to obese parents. The same tendency was seen among underweight parents who tended to misclassify their children’s weight status. Nevertheless, obese parents also showed a limitation to estimate their child’s weight status. Almost a third of obese parents with obese children misclassified their children as normal weight. This represents a public health concern, since parents provide food environments for their children’s early experiences with food and eating.

Parents also influence children’s own perceptions of their weight, and thus their recognition and response to education and opportunities to modify their diet and/or physical activity. Parental recognition and acceptance that their child is overweight is vital if interventions are to be initiated and successfully.

This is the first study conducted in a representative sample in Puerto Rico that explores perceptions of both children and parents about children’s weight status, particularly for overweight and obesity. This study showed a higher misperception of children’s overweight and obesity among less-educated parents compared to parents with higher education level. Therefore, it underscores the need for the design and development of education interventions targeted particularly to lower socio-economic families addressing the issues around overweight and obesity. Our results are consistent with the literature, reflecting that less-educated parents, children from less-educated families, children of overweight parents and parents of boys tend to underestimate overweight or obesity situations.

This study underscores the need to monitor children’s BMI for age and to share this information with parents so that tendencies toward overweight or obesity and even underweight could be identified on time. It is important to help parents understand their children’s weight status in order for parents to make better decisions for their children. It is also important to help children understand their nutritional and physiological needs in order to help them make better food selection and eat healthier.

Despite of these novel findings there were some limitations in our study. Although a considerably good response rate was obtained from children (63%), there was a low response rate from parents of children (44%), thus limiting comparison analyses among parental and children’s weight status and respective weight perceptions. Parental BMI was calculated from reported weight and height from parents which represents a limitation in terms of potential underreporting or imprecision of data provided. Children’s interviews were conducted in school settings which limited the time for the interviews, given the heavy workload of schools. Having more time for interviewing children would have given us more time to develop a more confident and truthful environment between the child and the interviewer in order to obtain more accurate responses from the child. Our study included very young age children who may have had limitations understanding and therefore assessing their own weight status.

In spite of these limitations, this research represents a significant contribution to the study of social factors associated to childhood obesity in Puerto Rico, since it assesses for the first time the perceptions of Puerto Rican parents about the weight status of their children. More importantly, our study brings an interesting dimension in this assessment when comparing parental perceptions with children’s own perceptions of children’s weight status. Studies confirm the influence that parents exert over children lifestyle characteristics, but to our knowledge, this is the first study that establishes an association among parents and children’s perceptions of weight conditions in a Hispanic population. A particular contribution is the finding that both parents and children with overweight and obesity had difficulties understanding children’s weight status. Thus, more attention should be given to help both parents and children to comprehend and accept their weight status in order to facilitate the adoption of healthy lifestyles.
